# Low Physical Activity Level and Short Sleep Duration Are Associated with an Increased Cardio-Metabolic Risk Profile: A Longitudinal Study in 8-11 Year Old Danish Children

**DOI:** 10.1371/journal.pone.0104677

**Published:** 2014-08-07

**Authors:** Mads F. Hjorth, Jean-Philippe Chaput, Camilla T. Damsgaard, Stine-Mathilde Dalskov, Rikke Andersen, Arne Astrup, Kim F. Michaelsen, Inge Tetens, Christian Ritz, Anders Sjödin

**Affiliations:** 1 Department of Nutrition, Exercise and Sports, Faculty of Science, University of Copenhagen, Copenhagen, Denmark; 2 Healthy Active Living and Obesity Research Group, Children’s Hospital of Eastern Ontario Research Institute, Ottawa, Ontario, Canada; 3 National Food Institute, Division of Nutrition, Technical University of Denmark, Søborg, Denmark; University of Rochester, United States of America

## Abstract

**Background:**

As cardio-metabolic risk tracks from childhood to adulthood, a better understanding of the relationship between movement behaviors (physical activity, sedentary behavior and sleep) and cardio-metabolic risk in childhood may aid in preventing metabolic syndrome (MetS) in adulthood.

**Objective:**

To examine independent and combined cross-sectional and longitudinal associations between movement behaviors and the MetS score in 8-11 year old Danish children.

**Design:**

Physical activity, sedentary time and sleep duration (seven days and eight nights) were assessed by accelerometer and fat mass index (fat mass/height^2^) was assessed using Dual-energy X-ray absorptiometry. The MetS-score was based on z-scores of waist circumference, mean arterial blood pressure, homeostatic model assessment of insulin resistance, triglycerides and high density lipoprotein cholesterol. All measurements were taken at three time points separated by 100 days. Average of the three measurements was used as habitual behavior in the cross-sectional analysis and changes from first to third measurement was used in the longitudinal analysis.

**Results:**

723 children were included. In the cross-sectional analysis, physical activity was negatively associated with the MetS-score (P<0.03). In the longitudinal analysis, low physical activity and high sedentary time were associated with an increased MetS-score (all P<0.005); however, after mutual adjustments for movement behaviors, physical activity and sleep duration, but not sedentary time, were associated with the MetS-score (all P<0.03). Further adjusting for fat mass index while removing waist circumference from the MetS-score rendered the associations no longer statistically significant (all P>0.17). Children in the most favorable tertiles of changes in moderate-to-vigorous physical activity, sleep duration and sedentary time during the 200-day follow-up period had an improved MetS-score relative to children in the opposite tertiles (P = 0.005).

**Conclusion:**

The present findings indicate that physical activity, sedentary time and sleep duration should all be targeted to improve cardio-metabolic risk markers in childhood; this is possibly mediated by adiposity.

## Introduction

Accelerometer-derived movement behaviors, such as moderate-to-vigorous physical activity (MVPA), sedentary time and sleep duration, have all been associated with cardio-metabolic risk in children [1]–[3]. Existing evidence in children linking these accelerometer-derived movement behaviors to cardio-metabolic risk is primarily cross-sectional, and combined associations have only recently been investigated between MVPA and sedentary time [4], [5], but not with sleep duration, despite its influence on cardio-metabolic risk [1]. Most studies use single cardio-metabolic risk markers as their outcome; however, a clustering of cardio-metabolic risk markers, producing a metabolic syndrome (MetS) score, is a more sensitive approach to identifying children with increased cardio-metabolic risk [6].

To our knowledge, no study to date has investigated independent and combined associations between accelerometer determined PA, sedentary time and sleep duration with several cardio-metabolic risk markers or a MetS-score in a cross-sectional or longitudinal study. As both objectively measured PA and MetS appear to track from childhood to adolescence and adulthood [7], [8], a better understanding of the relationships between movement behaviors (PA, sedentary time and sleep duration) and cardio-metabolic risk in childhood may aid in developing lifestyle interventions and public health policies to prevent the MetS in adulthood.

The aim of this paper was to examine independent and combined cross-sectional and longitudinal associations between objectively measured movement behaviors and cardio-metabolic risk markers, including the MetS-score, in a sample of Danish children. We hypothesized that high PA level, low sedentary time and long sleep duration would be independently associated with a lower MetS-score. Furthermore, we hypothesized that children in the most favorable tertiles of changes in MVPA, sedentary time and sleep duration during the follow-up period would have a more favorable development in the MetS-score than children in the opposite tertiles.

## Subjects and Methods

The initial sample comprised 834 of the 1,021 invited third and fourth grade students (8–11 years old) from nine Danish municipal schools enrolled in the OPUS (Optimal well-being, development and health for Danish children through a healthy New Nordic Diet) school meal study. The main aim of this cluster-randomized crossover study was to investigate the potential health effects of a New Nordic Diet served at school versus usual packed lunch (control) [9]. Measurements were performed at baseline (August to November 2011), before the end of the first dietary period (approximately 100 days later) and before the end of the second dietary period (after another 100 days). As the school meal intervention did not affect physical activity, sedentary time and sleep duration [10] all participants were used in the present paper regardless of randomization status. Average of the three measurements was used as habitual behavior in the cross-sectional analysis and changes from first to third measurement was used in the longitudinal analysis. Due to missing data [one or more of the five components of the MetS-score at baseline (n = 87), during the last visit (n = 91) and no measure of pubertal status (n = 24)], our cross-sectional and longitudinal samples comprised data from 723 and 632 children, respectively.

Questionnaire data were first collected, followed by simultaneous registrations of movement behaviors and dietary intake within the following two weeks, after which anthropometry, blood pressure and blood samples were collected the following week.

### Ethics Statement

The study was approved by the Committees on Biomedical Research Ethics for the Capital Region of Denmark (J.nr. H-1-2010-123). Child assent and written informed parental consent of both custody holders were obtained for all participants. The study was registered in the www.clinicaltrials.gov database (no. NCT01457794).

### Movement behaviors

The children were asked to wear an ActiGraph™ tri-axis accelerometer monitor (GT3X+ or GT3X, Pensacola, FL, USA) tightly on the right hip using an elastic belt for seven consecutive days and eight nights (entire 24-hour period); they were only allowed to remove it during water activities (i.e. showering or swimming). At the end of the observation period, data were reintegrated to 60-second epochs and analyzed using ActiLife6 (the ActiGraph 2012, ActiLife version 6). Before analysis of physical activity and sedentary time, we removed 1) data between midnight and six am, as this was expected to be non-awake time; 2) periods of at least 15 minutes of consecutive zero counts using tri-axial vector magnitudes to remove non-wear time and non-awake time; and 3) consecutive wear time periods of less than 60 minutes to remove non-awake time, as sleep for most children is characterized by minor periods of movement that we did not want to include in our analysis of physical activity and sedentary time. Total PA (counts/minute; cpm) was expressed as total vertical counts from monitor wear time divided by monitor wear time. As a secondary variable, total tri-axial PA (cpm) was expressed as a vector magnitude of the total tri-axial counts from monitor wear time, divided by monitor wear time. Time spent in a sedentary state was defined as all minutes showing 100 vertical cpm or less, which is a widely used cut-off point [11]. MVPA was defined as ≥2296 vertical cpm, which is a recently suggested pediatric cut-off point [11]. The percentage of time spent in a sedentary state was calculated by dividing sedentary time with monitor wear time and multiplying by 100. The number of children fulfilling the World Health Organization’s recommendation of an average of ≥60 minutes MVPA/day [12] was also calculated. The weekly averages of total PA, MVPA and sedentary time were calculated in the proportion of five to two between weekdays (Monday to Friday) and weekend days (Saturday and Sunday). Total PA, MVPA and sedentary time were only considered valid if monitor wear time was at least 10 hours/day for a minimum of three weekdays and one weekend day.

The parents and children were instructed to keep logs for bedtime (“lights off” and trying to sleep) and waking time (“lights on”) during the week in which the monitor was worn. Based on these self-reports, we calculated the proportion of children meeting the recommended sleep duration according to the guidelines of the National Sleep Foundation in the USA (≥10 hours/night) [13]. To estimate accelerometer determined sleep duration, the self-reported bedtimes and waking times were used as the possible window of sleep and accelerometer data within this window were scored in ActiLife6 using the algorithm by Sadeh *et al*. [14]. Self-reported sleep logs were missing for some children (98 sleep logs missing out of 1,638); in these cases, sleep was scored visually from the individual actograms as the difference between time when activity stopped and time when activity resumed. In a random subsample of 105 individuals, we found that the mean difference in sleep duration between these two approaches was small (3.8 minutes, P<0.001). The weekly average of sleep duration was calculated in the proportion of five to two between weekdays (Sunday to Thursday) and weekend days (Friday and Saturday). Sleep duration was only considered valid if it was measured for a minimum of three weekdays and one weekend day. On any of the three measurement occasions, ≥85% of individuals had valid activity and sleep registrations for seven days and nights.

### Anthropometry and cardio-metabolic risk markers

Clinical measurements and venous blood sampling from the antecubital vein were performed in the morning after an overnight fast in a mobile laboratory. Whole-blood glucose was analyzed immediately after sampling using a Hemocue Glucose 201 analyzer (Hemocue Danmark) and serum insulin, plasma triglycerides and plasma high density lipoprotein cholesterol (HDL-C) were analyzed according to a standard protocol described elsewhere [15]. Homeostatic model assessment of insulin resistance (HOMA_IR_) was calculated as plasma glucose (mmol/l) × serum insulin (mmol/l)/22.5 [16].

After 10 minutes of rest, blood pressure was measured three times in the supine position with an automated device (UA-787 Plus; A&D Medical) using two different cuff sizes (18–22 cm or 22–32 cm). A second device (ProBP 3400 Sure BP, Welch Allyn Inc., NY, USA) was used for children with arm circumferences <22 cm using three different cuff sizes (12–16 cm; 15–21 cm; and 20–26 cm). The mean of the last two measurements was used in the data analyses. The mean arterial blood pressure (MAP) was calculated as: 1/3 x systolic blood pressure +2/3 x diastolic blood pressure. Height (CMS Weighing Equipment LTD, London, UK) and waist circumference (position of the umbilicus) were measured three times to the nearest millimeter and the average was used. The children were weighed to the nearest 0.1 kg (Tanita BWB-800S, Tanita, Europe) while barefoot and wearing light clothing. Finally, whole-body composition was determined by Dual-energy X-ray absorptiometry (Lunar Prodigy; GE Medical Systems, Madison, Wisconsin, USA) using Encore software version 13.5 (Encore, Madison, WI, USA), and fat mass index (FMI) was calculated as fat mass divided by height squared. As FMI is relatively independent of fat-free mass [17], it was chosen over, for example, percentage of body fat.

The body mass index z-score was calculated based on the World Health Organization Growth Reference data from 2007 [18]. The prevalence of underweight, normal weight, overweight and obese children was calculated based on age- and sex-specific cut-offs defined to pass through a body mass index of 18.5, 25 and 30 kg/m^2^ at 18 years of age [19], [20]. The prevalence of non-normal triglycerides (>1.7 mmol/L) [21], HOMA_IR_ (>3.0) [22], blood pressure (>95th age-, sex- and height-specific percentile of systolic and/or diastolic pressure) [23] and HDL-C (<1.0) [21] were also calculated.

### Questionnaire data

A baseline questionnaire ascertained age, sex, school grade, highest education of the household (divided into four groups according to years of education: ≤10 years, 11–12 years, 13–16 years, ≥17 years), number of parents born in Denmark and screen time. Screen time was computed based on the parent-reported time that the child spent watching television, playing passive video games or using the computer for leisure activities on weekdays and weekend days. Time spent playing the Nintendo Wii or similar active video game devices was not included in screen time. The weekly average of screen time was calculated in the proportion of five to two between weekdays and weekend days. The number of children fulfilling the Canadian recommendation of less than two hours of screen time/day [24] was also calculated. Pubertal status was self-reported (parent and child) based on breast development among girls and pubic hair growth among boys on a scale from 1 to 5 [25]. A dichotomous variable indicating whether or not the child had entered puberty was used in the statistical analyses (1 or ≥2). Finally, the parents were asked to fill out the 33-item Children’s Sleep Habits Questionnaire (CSHQ) that screens for common sleep disturbances. On a three-point scale, parents reported the frequency of their child’s habits. Items were then summed, and higher scores denote the presence of sleep disturbances. The scale has previously demonstrated test-retest reliability and validity in school-aged children [26].

### Dietary assessment

Daily food and beverage intake was recorded over seven consecutive days using a Web-based Dietary Assessment Software for Children (WebDASC) tool that has been validated for fruits and vegetables [27]. Dietary intake was recorded at the end of each day using pictures of different portion sizes. Further description of the WebDASC is available elsewhere [28]. Changes in fat quality of the diet was used as a covariate in the analyses between movement behaviors and the MetS-score, as it has been proposed to be important in the etiology of cardiovascular diseases [29]. This was calculated as the ratio between saturated fatty acids (g/day) and total fatty acids (g/day). No individuals were excluded from the analytical sample due to a low (<1.05) or high (>2.29) reported energy intake, defined as energy intake divided by calculated basal metabolic rate [30].

### Statistical analysis

#### Cross-sectional analysis

Descriptive characteristics of the study sample were presented as mean and standard deviation (SD), median (interquartile range [IQR]) or as proportions. Sex differences were assessed using two-sample t-tests (variables were logarithmically transformed if they were not normally distributed) or Pearson’s chi-squared tests.

A linear mixed model with school and subject as random effects was used to test the association between habitual movement behaviors (average of baseline, day 100 and day 200) and cardio-metabolic risk markers (waist circumference, MAP, HOMA_IR_, triglycerides and HDL-C) on all three occasions (except for screen time and CSHQ, which were only obtained at baseline). HOMA_IR_ and triglycerides were positively skewed and were thus log-transformed. These analyses were adjusted for baseline age, sex, pubertal status, and sex-pubertal status interaction (Model 1). Model 2: as in Model 1+ mutual adjustments between MVPA, sedentary time and sleep duration; Model 3: as in Model 2+ fat mass index. The analysis of MAP was additionally adjusted for height.

The MetS-score variables (waist circumference, MAP, HOMA_IR_, triglycerides and HDL-C) were standardized one by one, using linear mixed models with school and subject as random effects, by regressing them onto age, sex, pubertal status and sex-pubertal status interactions, and the standardized residuals (z-scores) from all five risk markers were summed to obtain the corresponding MetS-score. The above mentioned Models 2 and 3 also apply to the MetS-score. Since HDL-C is inversely related to cardio-metabolic risk, it was multiplied by −1. A higher MetS-score indicates a less favorable cardio-metabolic risk profile. The selected MetS-score variables and demographic standardization was suggested by Eisenmann [6].

#### Longitudinal analysis

A linear regression model was used to evaluate associations between changes in movement behaviors and changes in cardio-metabolic risk markers during the 200-day period. These analyses were adjusted for baseline age, sex, pubertal status, sex-pubertal status interaction, days of follow-up, and the particular baseline movement behavior and baseline cardio-metabolic risk marker of interest (Model 1) [baseline MVPA and MAP is adjusted for in a model, including change in MVPA and MAP etc.]. Model 2: as in Model 1+ mutual adjustments between MVPA, sedentary time and sleep duration; Model 3: as in Model 2+ baseline and change in fat mass index.

Changes in MetS-score and movement behaviors were calculated as the value at day 200 minus the baseline value. The multiple linear regression analyses of the change in MetS-score were adjusted for a number of covariates (Model 1: days of follow-up, the particular baseline movement behavior of interest [baseline MVPA is adjusted for in a model including change in MVPA, etc.] and baseline MetS-score; Model 2: as in Model 1+ exposures mutually adjusted for each other [total PA, MVPA, sedentary time and sleep duration] where total PA was not adjusted for MVPA/sedentary time and vice versa; Model 3: as in Model 2+ waist circumference was removed from the MetS-score and baseline and changes in fat mass index were adjusted for). Removing waist circumference from the MetS-score and adjusting for adiposity is a recommended way to calculate a MetS-score excluding adiposity [31].

To assess whether combining movement behaviors provides synergistic associations, movement behavior variables were divided into tertiles using the first and third tertiles as the reference group and risk groups and presented as the differences in delta MetS-score (adjusted for days of follow-up) using a linear mixed model.

Results from the analyses were reported as partial correlation coefficients, unstandardized regression coefficients and 95% confidence intervals (CI). Linearity between residuals and the dependent variables in the model was visually checked, as were the assumptions of normality and variance homogeneity. Since there was no statistically significant sex interaction between baseline and changes in movement behaviors with the MetS-score, data for both sexes were pooled. The level of significance was set at P<0.05 and statistical analyses were conducted using STATA/IC 11.2 (Houston, USA).

## Results

### Cross-sectional analysis

The children’s baseline characteristics are shown in **Table 1**. Overall, 13% of the children were classified as overweight or obese and a total of 8% (n = 57), 6% (n = 46), 4% (n = 26) and 2% (n = 13) had non-normal blood pressure, HOMA_IR_, HDL-C and triglyceride levels, respectively. Only 27% (n = 190) of children accumulated at least 60 min of MVPA/day on average, 34% (n = 241) reported screen time to be no more than two hours/day and 45% (n = 303) reported sleep duration to be 10 hours/night or more.

**Table 1:** Baseline descriptive characteristics of the study population stratified by sex.

Variable	n	Boys (n = 374)	Girls (n = 349)	All (n = 723)
Demographics				
Age (years)	723	10.1±0.6	9.9±0.6 **	10.0±0.6
3^rd^ grade/4^th^ grade (%)	723	46/54	48/52	47/53
Tanner stage (% 1/2/≥3)	723	75.4/20.9/3.7	53.6/37.0/9.5 **	64.9/28.6/6.5
Parents born in Denmark (% 0/1/2)	723	8.6/13.1/78.3	8.3/12.3/79.4	8.4/12.7/78.8
Highest education of parents (%)^1^	721	4.6/32.7/39.7/23.1	5.8/36.2/38.5/19.5	5.1/34.4/39.1/21.4
Anthropometrics				
Weight (kg)	722	35.4±7.2	34.7±6.9	35.1±7.1
Height (cm)	723	142.8±7.1	142.1±7.0	142.4±7.1
Body mass index z-score^2^	722	0.21±1.11	0.06±1.04	0.14±1.08
Weight status (% uw/nw/ow/ob)^3^	722	9.1/77.5/11.2/2.1	11.8/74.7/11.8/1.7	10.4/76.2/11.5/1.9
Fat mass index (kg/height^2^)	720	3.1 (2.2;4.8)	4.1 (2.9;5.7) **	3.7 (2.5;5.3)
MetS-score markers				
Waist circumference (cm)	723	62.4 (59.3;68.2)	62.5 (58.9;68.1)	62.4 (59.3;68.2)
MAP (mm Hg)	723	80.9±6.0	81.5±6.6	81.2±6.3
HOMA_IR_	723	1.34 (0.98;1.89)	1.50 (1.09;2.09) *	1.40 (1.02;1.96)
Triglycerides (mmol/L)	723	0.57 (0.48;0.70)	0.66 (0.54;0.83) **	0.61 (0.50;0.77)
HDL-C (mmol/L)	723	1.49±0.32	1.39±0.29 **	1.44±0.31
Movement behaviors				
Total physical activity (cpm)^4^	697	520±138	453±118 **	487±133
Sedentary time (%)^5^	697	52.6±7.0	52.5±6.4	52.5±6.7
Screen time (min/day)	702	154 (114;210)	136 (94;183) **	148 (103;194)
MVPA (min/day)	697	57±24	38±16 **	48±23
Sleep duration (min/night)	679	553±27	556±28	554±27
CSHQ	719	42 (39;45)	42 (39;46)	42 (39;46)
Diet				
Energy intake (kJ/day)	708	8156±1721	7028±1456 **	7613±1694
SFA/Total fat	708	0.39±0.04	0.40±0.04	0.39±0.04

Abbreviations: MVPA, moderate-to-vigorous physical activity; CSHQ, Children’s Sleep Habits Questionnaire; MAP, mean arterial blood pressure; HOMA_IR_, homeostatic model assessment of insulin resistance; HDL-C, high density lipoprotein cholesterol; SFA, saturated fatty acid.

Data are presented as mean ± standard deviation, median (interquartile range) or proportions. Sex differences were tested using a two-sample t-test or Pearson’s chi-squared test: *, P<0.05; **, P<0.001.

1 Highest education of parents: ≤10 years/11–12 years/13–16 years/≥17 years.

2 Based on the World Health Organization Growth Reference from 2007 [18].

3 Based on age- and sex-specific cut-offs defined to pass through body mass index at 18.5, 25 and 30 kg/m^2^ at age 18 years [19], [20]; uw/nw/ow/ob, underweight/normal weight/overweight/obese. Data are presented as mean ± standard deviation or proportion.

4 Total tri-axial physical activity (cpm): Boys, 1052±232; Girls, 928±195 (P<0.001); All, 992±223.

5 Sedentary time (hh:mm/day): Boys, 7∶56±1∶07; Girls, 7∶51±1∶01 (P = 0.25); All, 7∶54±1∶04.

As shown in **Table 2**, all movement behaviors were associated with at least one of the five cardio-metabolic risk markers, but only total PA and MVPA were negatively associated with the MetS-score (all P<0.03) although not after mutual adjustment for movement behaviors and FMI (all P>0.72).

**Table 2:** Cross-sectional associations between movement behaviors and cardio-metabolic risk markers in Danish children.

	n	WC, cm	MAP, mm Hg^1^	HOMA_IR_ 2	Triglycerides, mmol/L^2^	HDL-C, mmol/L	MetS-score^3^
Total PA	564	−1.14 (−1.64;−0.64)** ¥	0.05 (−0.32;0.41)	−0.070 (−0.099;−0.042)* ¥ £	−0.036 (−0.057;−0.015)* ¥	0.034 (0.015;0.053)* ¥ £	−0.10 (−0.19;−0.01)*
MVPA	564	−0.93 (−1.23;−0.63)** ^¥£^	0.00 (−0.22;0.22)	−0.051 (−0.069;−0.034)** ¥	−0.029 (−0.042;−0.016)** ¥	0.023 (0.011;0.035)** ¥	−0.07 (−0.12;−0.01)*
Sedentary time	564	0.07 (−0.03;0.18)	−0.01 (−0.09;0.07)	0.007 (0.001;0.013)*	0.006 (0.002;0.010)*	−0.004 (−0.008;0.0005)	0.01 (−0.01;0.03)
Screen time	702	0.33 (−0.07;0.73)	0.22 (−0.12;0.56)	0.026 (0.000;0.053)*	0.012 (−0.008;0.031)	−0.002 (−0.019;0.014)	0.02 (−0.10;0.15)
Sleep duration	473	−2.21 (−3.79;−0.62)* ¥	−0.58 (−1.74;0.58)	−0.080 (−0.174;0.014) ¥	−0.022 (−0.089;0.046)	0.001 (−0.063;0.066)	−0.12 (−0.43;0.18)
CSHQ	719	0.16 (0.08;0.25)**	0.10 (0.02;0.17)*	0.007 (−0.002;0.013) *	0.006 (0.002;0.010)* £	−0.001 (−0.005;0.002)	0.02 (−0.01;0.05)

Abbreviations: PA, physical activity; MVPA, moderate-to-vigorous physical activity; CSHQ, Children’s Sleep Habits Questionnaire; WC, waist circumference; MAP, mean arterial blood pressure; HOMA_IR_, homeostatic model assessment of central insulin resistance; HDL-C, high density lipoprotein cholesterol; MetS-score, metabolic syndrome score.

Data are presented as unstandardized regression coefficients (β) with 95% confidence intervals (CI) using a linear mixed model with school and subject as random effects. The five cardio-metabolic risk markers were adjusted for baseline age, sex, pubertal status, and sex-pubertal status interaction (**Model 1**).

Coefficients represent the change in the outcome for a 100-cpm change in total PA, a 10-minute change in time spent in MVPA, a 1% change in sedentary time, a 60-minute change in screen time and sleep duration and a 1-point change in CSHQ. *, P<0.05; **, P<0.001.

¥ P<0.05 in **Model 2**: Model 1+ mutual adjustments between MVPA, sedentary time and sleep duration. Total PA was only adjusted for sleep duration.

£ P<0.05 in **Model 3**: Model 2+ fat mass index.

1 MAP was also adjusted for height.

2 HOMA_IR_ and triglycerides were log transformed.

3 MetS-score = (z-scores by baseline age, sex, pubertal status and sex-pubertal status interaction of) WC + MAP + HOMA_IR_ + triglycerides – HDL-C.

### Longitudinal analysis

As shown in **Table 3**, changes in total PA, MVPA and sedentary time during the 200 days were all associated with changes in HDL-C (P<0.001) and HOMA_IR_ (all P<0.02). Additionally, the change in MVPA was negatively associated with the change in triglycerides (P = 0.01), and changes in sleep duration was negatively associated with changes in HOMA_IR_ only after adjusting for PA and sedentary time (P = 0.02).

**Table 3:** Associations between changes in movement behaviors and changes in cardio-metabolic risk markers over a 200-day follow-up period in Danish children.

	n	WC, cm	MAP, mm Hg^1^	HOMA_IR_	Triglycerides, mmol/L	HDL-C, mmol/L
Total PA	554	−0.15 (−0.31;0.01)	0.03 (−0.26;0.33)	−0.07 (−0.13;−0.02)* ¥	−0.02 (−0.04;0.004)	0.029 (0.019;0.039)** ¥ £
MVPA	554	−0.12 (−0.23;0.0003)	−0.001 (−0.23;0.21)	−0.07 (−0.11;−0.03)** ¥	−0.02 (−0.04;−0.004)*	0.019 (0.012;0.026)** ¥ £
Sedentary time	554	0.01(−0.03;0.05)	0.0002 (−0.08;0.08)	0.02 (0.003;0.03)*	0.003 (−0.003;0.009)	−0.006 (−0.009;−0.004)** ¥ £
Sleep duration	486	−0.10 (−0.67;0.46)	−0.51 (−1.49;0.47)	−0.18 (−0.36;0.01) ¥ £	−0.03 (−0.11;0.06)	0.003 (−0.034;0.039)

Abbreviations: PA, physical activity; MVPA, moderate-to-vigorous physical activity; WC, waist circumference; MAP, mean arterial blood pressure; HOMA_IR_, homeostatic model assessment of central insulin resistance; HDL-C, high density lipoprotein cholesterol.

Data are presented as unstandardized regression coefficients (β) with 95% confidence intervals (CI) using a linear mixed model with school as random effect. **Model 1:** Adjusted for baseline age, sex, pubertal status, sex-pubertal status interaction, days of follow up, and the particular baseline movement behavior and baseline cardio-metabolic risk component of interest. Coefficients represent the change in the outcome for a 100-cpm change in total PA, a 10-minute change in time spent in MVPA, a 1% change in sedentary time and a 60-minute change in sleep duration.*, P<0.05; **, P<0.001.

¥ P<0.05 in **Model 2**: Model 1+ mutual adjustments between MVPA, sedentary time and sleep duration. Total PA was only adjusted for sleep duration.

£ P<0.05 in **Model 3**: Model 2+ baseline and change in fat mass index.

1 MAP was also adjusted for baseline and change in height.

As reported in **Table 4**, low total PA, low MVPA and high sedentary time were associated with an increased MetS-score (all P<0.005) (*Model 1*). After mutual adjustment for the other movement behaviors, low total PA, low MVPA and short sleep duration were associated with an increased MetS-score (all P<0.03) (*Model 2*). Adjusting for saturated-to-total fatty acids attenuated these prospective associations and were no longer significant (P = 0.054 to 0.07). Adjusting for FMI while removing waist circumference from the MetS-score rendered the associations no longer significant (all P>0.17) (*Model 3*).

**Table 4:** Associations between changes in movement behaviors and changes in the metabolic syndrome (MetS) score^1^ over a 200-day follow-up period in Danish children.

		Model 1 (n = 486–554)			Model 2 (n = 469)			Model 3 (n = 465)		
Variable	N	r	β (95% CI)	P	r	β (95% CI)	P	r	β (95% CI)	P
Total PA^2^	554	−0.13	−0.18 (−0.29;−0.07)	**0.002**	−0.11	−0.14 (−0.25;−0.02)	**0.02**	−0.03	−0.03 (−0.14;0.07)	0.51
MVPA	554	−0.18	−0.18 (−0.27;0.10)	**<0.001**	−0.10	−0.12 (−0.22;−0.01)	**0.03**	−0.03	−0.03 (−0.13;0.06)	0.48
Sedentary time	549	0.12	0.04 (0.01;0.07)	**0.005**	0.04	0.02 (−0.02;0.06)	0.39	0.04	0.02 (−0.02;0.05)	0.39
Sleep duration	486	−0.06	−0.25 (−0.64;0.15)	0.22	−0.10	−0.46 (−0.87;−0.04)	**0.03**	−0.07	−0.26 (−0.63;0.11)	0.17

Abbreviations: PA, physical activity; MVPA, moderate-to-vigorous physical activity.

Data are presented as partial correlation coefficients (*r*), unstandardized regression coefficients (β) and 95% confidence intervals (CI) using a linear mixed model with school as random effect. Coefficients represent the change in MetS-score for a 100-cpm change in total PA, a 10-minute change in time spent in MVPA, a 1% change in sedentary time and a 60-minute change in sleep duration. **Model 1**: The MetS variables were standardized by age, sex, pubertal status and sex-pubertal status interaction and the MetS-score was adjusted for days of follow up, the particular baseline movement behavior of interest and baseline MetS-score. **Model 2**: Model 1+ mutual adjustments between MVPA, sedentary time and sleep duration. Total PA was only adjusted for sleep duration. **Model 3**: Model 2+ waist circumference was removed from the MetS-score and baseline and changes in fat mass index was adjusted for.

1 MetS-score = (z-scores by baseline age, sex, pubertal status and sex-pubertal status interaction of) waist circumference + MAP + HOMA_IR_ + triglycerides – HDL-C.

2 Total tri-axial physical activity (100-cpm), Model 1: [r = −0.15, β = −0.14, P<0.001], Model 2: [r = −0.12, β = −0.11, P = 0.01], Model 3: [r = −0.04, β = −0.03, P = 0.41].

**Figure 1:**
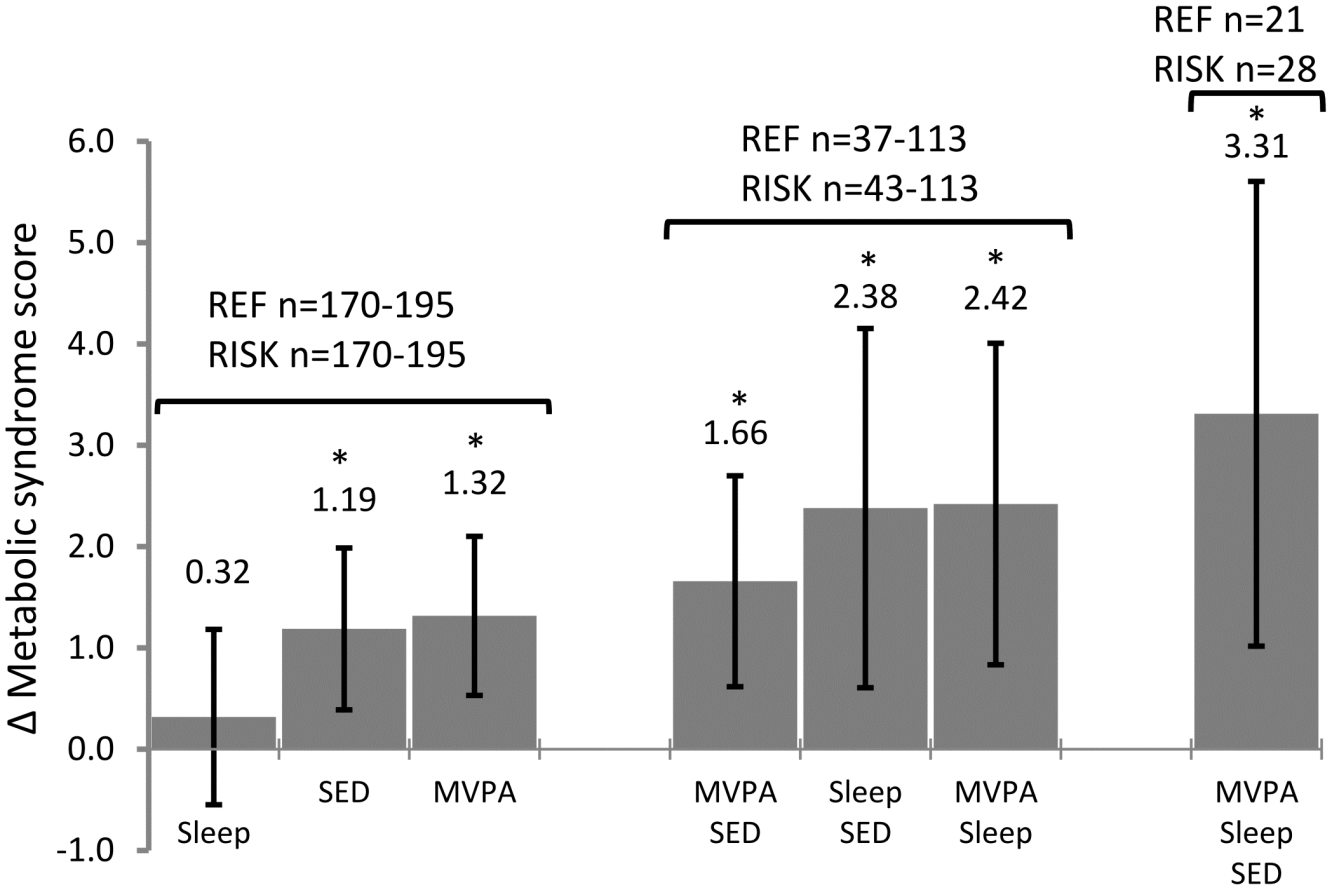
Combined associations between changes in movement behaviors and changes in the metabolic syndrome (MetS) score^1^ over a 200-day follow-up period in Danish children. Change in movement behaviors between baseline and day 200 was divided into tertiles using the 1^st^ and 3^rd^ tertile as a reference group (REF) and a risk group (RISK). Data are presented as unstandardized regression coefficients (β) with 95% confidence intervals (CI) between reference group and risk group using a linear mixed model. Sleep, sleep duration (RISK<−24.8 vs. REF>−5.5 min/night); SED, sedentary time (RISK>1.6 vs. REF<−3.4%); MVPA, moderate-to-vigorous physical activity (RISK<0.3 vs. REF>15.2 min/day). Adjusted for days of follow up ^1^MetS-score = (age, sex, pubertal status and sex-pubertal status interaction standardised z-scores of) waist circumference+MAP+HOMA_IR_+triglycerides – HDL-C. *P = 0.001 to 0.009.

The combined associations between tertiles of changes in movement behaviors and the MetS-score during the follow-up period are shown in **Figure 1**. Children in the most favorable tertiles of changes in MVPA, sleep duration and sedentary time had a decrease in the MetS-score of 3.31 (95% CI 1.02;5.61, P = 0.005) relative to children in the opposite tertiles. Mean (SD) change in the MetS-score was −0.04 (3.82). The change in MetS-score between the most favorable and least favorable tertile of movement behavior was due to a difference of 0.54 (95% CI 0.001;1.08; P = 0.049) in HOMA_IR_ and −0.14 mmol/L (95% CI −0.24;−0.04, P = 0.008) in HDL-C but no differences in waist circumference [1.0 cm (95% CI −0.5;2.4, P = 0.19)], triglycerides [0.15 mmol/L (95% CI −0.08;0.37, P = 0.20)] and MAP [0.52 mm Hg (95% CI −2.86;3.89, P = 0.76)].

Of interest, all significant findings between movement behaviors and the MetS-score remained significant (and insignificant associations remained insignificant) if not standardized according to baseline age, sex, pubertal status and their interaction or after further adjusting for the number of parents born in Denmark and highest education of the household.

## Discussion

Collectively, we observed that low habitual total PA and MVPA were associated with a higher MetS-score in our cross-sectional sample. In the longitudinal analysis, low total PA, low MVPA and short sleep duration were associated with an increased MetS-score after mutual adjustment for other movement behaviors; however, after adjustment for FMI, the associations became non-significant, suggesting that adiposity could be a mediator of these relationships. Although sedentary time was not independent of MVPA, synergistic effects between movement behaviors and the MetS-score were observed in combined associations. The present findings are interesting and suggest that multiple movement behaviors should be targeted to reduce cardio-metabolic risk in childhood. They also suggest that this is possibly mediated by adiposity.

Results from this study support previous cross-sectional observations showing that higher total PA and MVPA are associated with a healthier cardio-metabolic profile in children and adolescents [2], [4], [5], [31] and that higher sedentary time and/or screen time are associated with a less healthy cardio-metabolic profile [3]–[5], [32]–[34], at least in subgroups of the population [35]. When mutually adjusting PA and sedentary time for each other, associations between cardio-metabolic risk and PA often remain but attenuate between cardio-metabolic risk and sedentary time [4], [5], [31], although accelerometer-determined sedentary time has been positively associated with clustered cardio-metabolic risk independent of total PA [36]. Studies tend to find stronger associations between screen time and cardio-metabolic risk markers than between accelerometer-determined sedentary time and cardio-metabolic risk markers [4], [32], suggesting screen time to be more detrimental than non-screen sedentary behaviours in relation to cardio-metabolic risk. Reasons for this could be that children tend to eat in the absence of hunger while engaged in such activities [37] and possibly also decrease sleep duration.

In the present study, the only cardio-metabolic risk marker associated with sleep duration was waist circumference as well as HOMA_IR_ after adjusting for PA and sedentary time, whereas self-reported sleep disturbances were associated with several risk markers. However, none of these sleep measures were associated with the MetS-score in our cross-sectional analysis. Another study using accelerometers in obese adolescents found no association between sleep duration and the prevalence of metabolic syndrome [38]. Other studies using accelerometer-determined sleep duration and quality found a negative association with blood pressure [39] or no association [40], whereas evidence for a link between short sleep duration and insulin resistance [41]–[45] or waist circumference [46] is more evident.

To our knowledge, no study has assessed changes in accelerometer-determined sedentary time or sleep duration with changes in a clustered cardio-metabolic risk score in children, and only a single study has assessed this for PA finding no prospective association between MVPA and the MetS-score, but an inverse association between vigorous PA and the MetS-score [47]. Results from exercise interventions have reported favourable changes in waist circumference, HDL-C, triglycerides and insulin levels in obese children [48] and reduced systolic blood pressure in normotensive adolescents [49]. Although we did not find changes in total PA and MVPA to be associated with all of these cardio-metabolic risk markers, we found that increased total PA and MVPA were associated with decreased MetS-score. The prospective association between sedentary time and MetS-score disappeared after adjusting for MVPA; however, MVPA and sleep duration were independently associated with the MetS-score even after adjusting for sedentary time.

Quality of fat intake has been proposed to be important in the etiology of cardiovascular diseases [29] and intake of saturated fatty acids has been found to be positively associated with insulin levels and systolic blood pressure in children [50]; however, adjusting for intake of dietary fat quality only changed the beta-coefficients slightly. Instead, the prospective associations between movement behaviours and the MetS-score became non-significant after controlling for FMI, indicating that adiposity partly explains the reported associations. In another large cross-sectional study of children and adolescents, the association between PA and clustered cardio-metabolic risk was found to be independent of skinfold thickness [32]. The authors suggest that their precise measurement of activity (accelerometers) explains why they were able to show an association between activity and CVD risk independent of fatness [51]; however, this could also have been caused by the suboptimal adiposity measure. A recent study supports our finding as their cross-sectional association between PA/sedentary time and insulin sensitivity disappeared after controlling for adiposity [52].

In a recent cross-sectional study of 536 children with at least one obese parent, waist circumference and diastolic blood pressure were lower and HDL-C higher among children in the upper tertile of MVPA and lower tertile of sedentary time when compared to the opposite (P<0.01) [4]. Likewise, a large cross-sectional study of approximately 20,000 children and adolescents, using the same tertiles of MVPA and sedentary time (combined associations), found the expected differences in waist circumference, systolic blood pressure, insulin, triglycerides and HDL-C (P<0.05) [5]. This was despite the fact that some markers (especially insulin) appeared to be better with higher sedentary time across all MVPA tertiles. As these studies did not compare tertiles of MVPA and sedentary time separately, it is difficult to evaluate the potential synergistic effect when combined. We did so in our longitudinal analyses and found a gradual, although not significant, increase in the MetS-score when going from one to three movement behaviors (sleep duration, MVPA and sedentary time). Therefore, despite the fact that sedentary time and MVPA were not independently associated with the MetS-score, combined associations were evident between sedentary time/MVPA/sleep duration and the MetS-score. Our data, therefore, suggest that multiple movement behaviors should be targeted to improve cardio-metabolic risk markers in childhood.

The magnitude of the cross-sectional associations between movement behaviors and cardio-metabolic risk markers were relatively small, which is most likely because movement behaviors are highly variable in children [10] and because our measurements are only proxies of the behaviors we are trying to assess. Moreover, the children are mainly healthy; however, waist circumference, triglycerides and HDL-C had higher beta-values than what was observed in two recent studies [4], [5], despite the fact that our children were less obese. The most likely explanation for this is that our use of an average of three seasons removed some of the within-person variability (including season specific differences and longitudinal trends) and thereby got better estimates of habitual behavior. Results from the combined analyses in movement behaviors were more substantial. Children in the tertiles with the most favorable changes in MVPA, sleep duration and sedentary time during the follow-up period compared with those in the least favorable tertiles of changes had remarkably lower HOMA_IR_ and higher HDL-C. These differences are comparable to what has been observed from a successful 12-week aerobic exercise program for obese children [48]. The magnitude of changes observed in cardio-metabolic risk markers due to healthy movement behaviors in our study, therefore, seems to be meaningful from a public health standpoint.

The strength of our study included repeated measures of a large and well-characterized sample of children (demographics, diet, accelerometer-determined movement behaviors and FMI) that allowed us to adjust for a number of variables when looking at changes occurring over time. Another strength is the MetS-score, which mainly stems from the fact that there is no clear definition of the MetS in children or adolescents and that the prevalence rate is relatively low. The approach is more sensitive and statistically less prone to errors compared to the dichotomous approach. However, sample-specific values cannot be compared to other studies and cannot determine the prevalence of MetS. Strengths and limitations of the accelerometer-derived movement behaviours including attachment site, epoch length, cut-points, season variation and the possible influence of cycling, have been discussed previously [10], [53], [54]. Even though we used a longitudinal approach, we cannot determine cause and effect associations, although it is unlikely that the cardio-metabolic risk markers led to impaired movement behaviors, except from adiposity [5], [54].

In summary, low MVPA and short sleep duration were associated with an increased cardio-metabolic risk profile over a 200-day follow-up period in a sample of Danish children. Furthermore, simultaneous unfavorable changes in MVPA, sedentary time and sleep duration synergistically increased the cardio-metabolic risk profile of children. The present findings indicate that multiple movement behaviors should be targeted to improve cardio-metabolic risk markers in childhood and that this is possibly mediated by adiposity.
